# Supplementary material for: Differential Role of Type 2 Diabetes as a Risk Factor for Tuberculosis in the Elderly versus Younger Adults
Source: Pathogens. 2022 Dec 16;11(12):1551. doi: 10.3390/pathogens11121551 (PMC9784720; doi:10.3390/pathogens11121551)
Supplement: Supplementary file 1 [file pathogens-11-01551-s001.zip › pathogens-2051015-supplementary.pdf]

Table S1. P values for associations between TB status and each of the host factors shown in Table 1, by age group *

	All (≥ 18 y/o) p value	YA (18-44 y/o) p value	MAA (45-64 y/o) p value	ELD (≥ 65 y/o) p value
**Sociodemographics**				
**Male sex**	**<0.001**	**<0.001**	**<0.001**	**<0.001**
**Education, High school or higher**	**<0.001**	**<0.001**	0.518	0.562
**Current or past smoker**	**<0.001**	**<0.001**	**0.007**	**0.036**
**TB-related variables**				
**Recent TB exposure (ReC vs CoC)**				
**Past TB**	**<0.001**	**0.008**	0.162	**0.027**
**BCG**	**<0.001**	**0.078**	0.295	**0.002**
**Type 2 diabetes and other conditions**				
**Type 2 diabetes**	**<0.001**	**<0.001**	**<0.001**	0.321
**Overweight/obese, BMI ≥ 25**	**<0.001**	**<0.001**	**<0.001**	**<0.001**
**Central obesity (M ≥ 0.90 M; F ≥0.86)**	**<0.001**	**<0.001**	**0.001**	0.313
**High cholesterol (200 mg/dL)**	**<0.001**	**0.069**	**<0.001**	**<0.001**
**High LDL (100 mg/dL)**	**<0.001**	**0.001**	**<0.001**	**<0.001**
**Low HDL (40 M, 50 F, mg/dL)**	0.607	0.866	0.266	0.748
**High Triglycerides (150 mg/dL)**	**<0.001**	**<0.001**	**<0.001**	**<0.001**
**Macrovascular diseases**	0.234	0.282	**0.052**	**0.014**
**Microvascular diseases**	**<0.001**	**0.008**	**<0.001**	0.356
**Anti-inflammatory medications**	**0.001**	**0.001**	0.131	0.823

* Data from Table 1 was used to identify associations between host characteristics and TB status among all participants or by age group. Analysis was done by chi-square or Fisher's exact; p ≤ 0.099 shown in bold; Normal range values shown in parenthesis; M, males; F, females.

Table S2. Multivariable logistic regression models for the association between diabetes (exposure) and TB (outcome) by age group *

	All (≥ 18 y/o)	YA (18-44 y/o)	MAA (45-64 y/o)	ELD (≥ 65 y/o)
**Models controlling for core variables associated with TB outcomes in all age groups as shown in Table S1**				
**Diabetes (vs no diabetes)**	4.12 (2.86, 5.93)	6.48 (3.35, 12.53)	5.4 (2.95, 9.89)	1.87 (0.75, 10.95)
**Male sex (vs female)**	3.4 (2.34, 4.94)	2.79 (1.58, 4.91)	4.34 (2.37, 7.92)	4.03 (1.48, 46.78)
**BMI, (UW/NOR vs OW/OB)**	12.03 (8.31, 17.42)	18.97 (10.28, 35.01)	7.82 (4.38, 13.95)	16.22 (5.63, 4.14)
**Current or past smoker (vs never)**	1.51 (1.03, 2.22)	1.1 (0.61, 2)	2.05 (1.13, 3.74)	1.46 (0.52, 0)
**Sensitivity analysis. Models controlling for variables associated with TB depending on the age group, as shown in Table S1**				
**Diabetes (vs no diabetes)**	3.64 (2.51, 5.29)	5.3 (2.65, 10.62)	5.19 (2.79, 9.67)	1.81 (0.72, 43.09)
**Male sex (vs female)**	3.47 (2.36, 5.1)	2.73 (1.51, 4.96)	4.65 (2.47, 8.75)	3.78 (1.38, 45.81)
**BMI, (UW/NOR vs OW/OB)**	12.04 (8.24, 17.59)	19.46 (10.37, 36.53)	7.82 (4.29, 14.26)	14.78 (5.07, 10.34)
**Current or past smoker (vs never)**	1.51 (1.02, 2.24)	1.07 (0.57, 2.01)	2.16 (1.17, 3.98)	1.46 (0.52, 43.09)
**Lower education (vs HS or higher)**	1.52 (1.03, 2.23)	1.96 (1.13, 3.4)	1.48 (0.77, 2.85)	NT
**BCG at birth (vs none)**	NT	NT	NT	0.56 (0.19, 45.81)
**Pain/NSAID use (vs none)**	1.94 (1.29, 2.9)	3.05 (1.59, 5.84)	1.69 (0.88, 3.27)	NT

* Data expressed as adjusted odds ratio (95% CI) after controlling for the variables analyzed for each model; BMI, body-weight index; UW/NOR, underweight or normal weight; OW/OB, overweight or obese; Pain/NSAID, report use of pain medication, including non-steroidal anti-inflammatory drug (NSAID) use in past month; NT, variable not entered into the model because p value above 0.100 by univariable analysis in the corresponding age group; HS, High school degree

Table S3. Unique characteristics of diabetes patients without TB for laboratory data, by age groups

	All (≥ 18 y/o)		YA (18-44 y/o)		MAA (45-64 y/o)		ELD (≥ 65 y/o)		
		**n=241**		**n=60**		**n=134**		**n=47**	
**Lipids (mg/dL)**	**n**	**%**	**n**	**%**	**n**	**%**	**n**	**%**	**p value**
High cholesterol (200)	68	28.2%	16	26.7%	40	29.9%	12	25.5%	0.822
Low HDL (40 M, 50 F)	179	74.3%	49	81.7%	97	72.4%	33	70.2%	0.306
High LDL (100)	126	52.3%	28	46.7%	76	56.7%	22	46.8%	0.459
High Triglycerides (150)	110	45.6%	27	45.0%	65	48.5%	18	38.3%	0.478
**Complete Blood Counts (x10e3/µL)**	**n**	**median (IQR)**	n	**median (IQR)**	n	**median (IQR)**	n	**median (IQR)**	**p value**
Platelets (146-388)	203	252 (95.00)	49	**253 (101.00)**	111	**264 (85.00)**	43	217 (71.00)	**<0.001**
Eosinophils (0.0-0.4)	213	0.17 (0.17)	50	0.18 (0.21)	119	**0.16 (0.14)**	44	0.21 (0.24)	**0.087**
Lymphocytes (0.8-3.3)	213	2.13 (0.86)	50	2.17 (0.92)	119	2.15 (0.87)	44	2.03 (0.72)	0.380
Monocytes (0.2-1.0)	213	0.45 (0.17)	50	0.46 (0.15)	119	0.45 (0.18)	44	0.45 (0.22)	0.799
Neutrophils (2.3-7.7)	213	4.29 (1.90)	50	**4.66 (1.76)**	119	**4.28 (1.76)**	44	3.93 (1.73)	**0.026**
White blood cells (4.8-10.9)	213	7.20 (2.40)	50	**7.55 (3.40)**	119	**7.30 (2.00)**	44	6.65 (2.40)	**0.050**
Hemoglobin (11.6-15.9 mg/dL)	204	13.70 (1.70)	49	**13.90 (1.80)**	111	**13.70 (1.60)**	44	13.20 (2.00)	**0.005**
**Oxidation status and Vitamin D**	**n**	**median (IQR)**	n	**median (IQR)**	n	**median (IQR)**	n	**median (IQR)**	**p value**
Reduced:oxidized glutathione ratio	97	0.58 (0.10)	21	0.58 (0.09)	45	0.58 (0.14)	31	0.60 (0.09)	0.752
Vitamin D levels (ng/mL)	98	26.09 (11.14)	18	24.59 (8.80)	53	24.59 (10.29)	27	29.09 (13.58)	0.333

Data expressed as column % for categorical variables or median (interquartile range, IQR) for continuous; Normal range values for each parameter shown in parenthesis; M, males; F, Females; p values ≤ 0.099 shown in bold; Bold font in young or middle age adults indicates significant or borderline significant differences when compared to elderly group. YA, young adults; MAA, middle age adults; ELD, elderly.

Table S4. Extended analysis of data from Table 2: Crude and adjusted p values for host characteristics associated with the ELD age group, in T2D-No TB participants

	YA vs ELD	MAA vs ELD	YA vs ELD	MAA vs ELD	YA vs ELD	MAA vs ELD
	Crude p		Adj p (Sex, SFU)		Adj p (Sex, NSAID)	
**Sociodemographics, medications and TB-related characteristics**						
**Male sex**	**0.047**	0.732				
**Education, High school or higher**	**<0.001**	**0.002**	**<0.001**	0.197	**<0.001**	0.186
**Current smoker**	0.161	0.659	0.449	0.386	0.327	0.431
**NSAID use**	**0.018**	0.394	**0.038**	0.477		
**BCG vaccination**	0.594	0.616	0.549	0.825	0.927	0.481
**Latent TB infection**	0.671	0.828	0.487	0.489	0.697	0.732
**Diabetes history**						
**Family history of diabetes**	**0.064**	0.115	0.059	0.645	0.253	0.419
**Self-reported diabetes**	0.261	0.951	0.784	0.516	0.569	0.549
**Years with diabetes, yrs**	**0.001**	**0.021**	**0.001**	**0.002**	**0.001**	**0.002**
**Glucose management**						
**Hyperglycemia (125 mg/dL)**	**0.014**	**0.041**	0.114	0.329	0.192	0.426
**HbA1c (%)**	**0.037**	0.131	**0.002**	**0.020**	**0.010**	**0.058**
**Glycemic Index (HbA1c*T2Dears)**	**0.009**	**0.073**	**0.041**	**0.095**	**0.015**	**0.076**
**Insulin Levels (mU/L)**	0.158	0.339	0.741	0.187	0.413	0.325
**HOMA-IR (Median, IQR)**	**0.002**	**0.002**	**0.001**	**0.004**	**0.001**	**0.005**
**Diabetes medications in past month**						
**Any**	**0.052**	**0.793**	**0.702**	**0.364**	**0.213**	**0.369**
**Insulin**	0.276	0.303	0.330	0.634	0.704	0.571
**Metformin**	0.304	0.383	0.882	**0.086**	0.424	**0.072**
**Sulfonylureas**	**0.001**	0.127			**0.007**	0.481
**Metformin + Sulfonylureas**	**0.021**	0.572			**0.056**	0.241
**Diabetes-associated conditions**						
**Body-mass index**	**0.009**	0.305	**0.031**	0.328	**0.023**	0.297
**Central obesity** (M ≥ 0.90 M; F ≥0.86)	**0.037**	**0.005**	0.255	0.083	0.444	**0.090**
**Macrovascular diseases**	**<0.001**	**0.004**	**<0.001**	0.862	**<0.001**	0.968
**Microvascular diseases**	0.218	0.231	0.770	0.491	0.208	0.823

* Data from Table 2 was used to expand analysis on associations between host characteristics and age groups in T2D-no TB participants. P values calculated by chi-square or Fisher's exact (crude p), multivariable logistic

regression (Proc LOGISTIC in SAS; categorical variables) or generalized linear models (proc GENMOD in SAS; continuous variables); $p \leq 0.099$ shown in bold; Cut-off values shown in parenthesis; M, males; F, females.

Table S5. Extended analysis of data from Table S3: Crude and adjusted p values for laboratory characteristics associated with the ELD age group, in T2D-No TB participants

	YA vs ELD	MAA vs ELD	YA vs ELD	MAA vs ELD	YA vs ELD	MAA vs ELD
	Crude p		Adj p (Sex, SFU)		Adj p (Sex, NSAID)	
**Lipids (mg/dL)**						
High cholesterol (200)	0.947	0.627	0.897	0.623	0.784	0.428
Low HDL (40 M, 50 F)	0.906	0.911	0.193	0.477	**0.077**	0.353
High LDL (100)	0.226	0.257	0.517	0.133	0.259	0.076
High Triglycerides (150)	0.486	0.243	0.735	0.294	0.784	0.299
**Complete Blood Counts (x10e3/μL)**						
Platelets (146-388)	**0.005**	**<0.001**	**0.001**	**<0.001**	**0.001**	**<0.001**
Eosinophils (0.0-0.4)	0.249	**0.031**	**0.064**	**0.001**	0.100	**0.002**
Lymphocytes (0.8-3.3)	0.196	0.236	**0.057**	0.100	**0.045**	**0.091**
Monocytes (0.2-1.0)	0.916	0.652	0.766	0.335	0.982	0.376
Neutrophils (2.3-7.7)	**0.010**	**0.071**	**0.007**	**0.044**	**0.003**	**0.038**
White blood cells (4.8-10.9)	**0.022**	**0.066**	**0.016**	0.086	**0.007**	**0.070**
Hemoglobin (11.6-15.9 mg/dL)	**0.003**	**0.012**	**0.003**	**0.004**	**0.002**	**0.003**
**Oxidation status and Vitamin D levels**						
Reduced to oxidized glutathione ratio	0.830	0.450	NS	NS	0.722	0.221
Vitamin D levels (ng/mL)	0.276	0.156	NS	NS	0.431	0.335

Data from Table S3 was used to expand analysis on associations between host laboratory characteristics and age groups in T2D-no TB participants. P values calculated by chi-square or Fisher's exact (crude p), multivariable logistic regression (Proc LOGISTIC in SAS; categorical variables) or generalized linear models (proc GENMOD in SAS; continuous variables); p ≤ 0.099 shown in bold; Cut-off values shown in parenthesis; M, males; F, females.

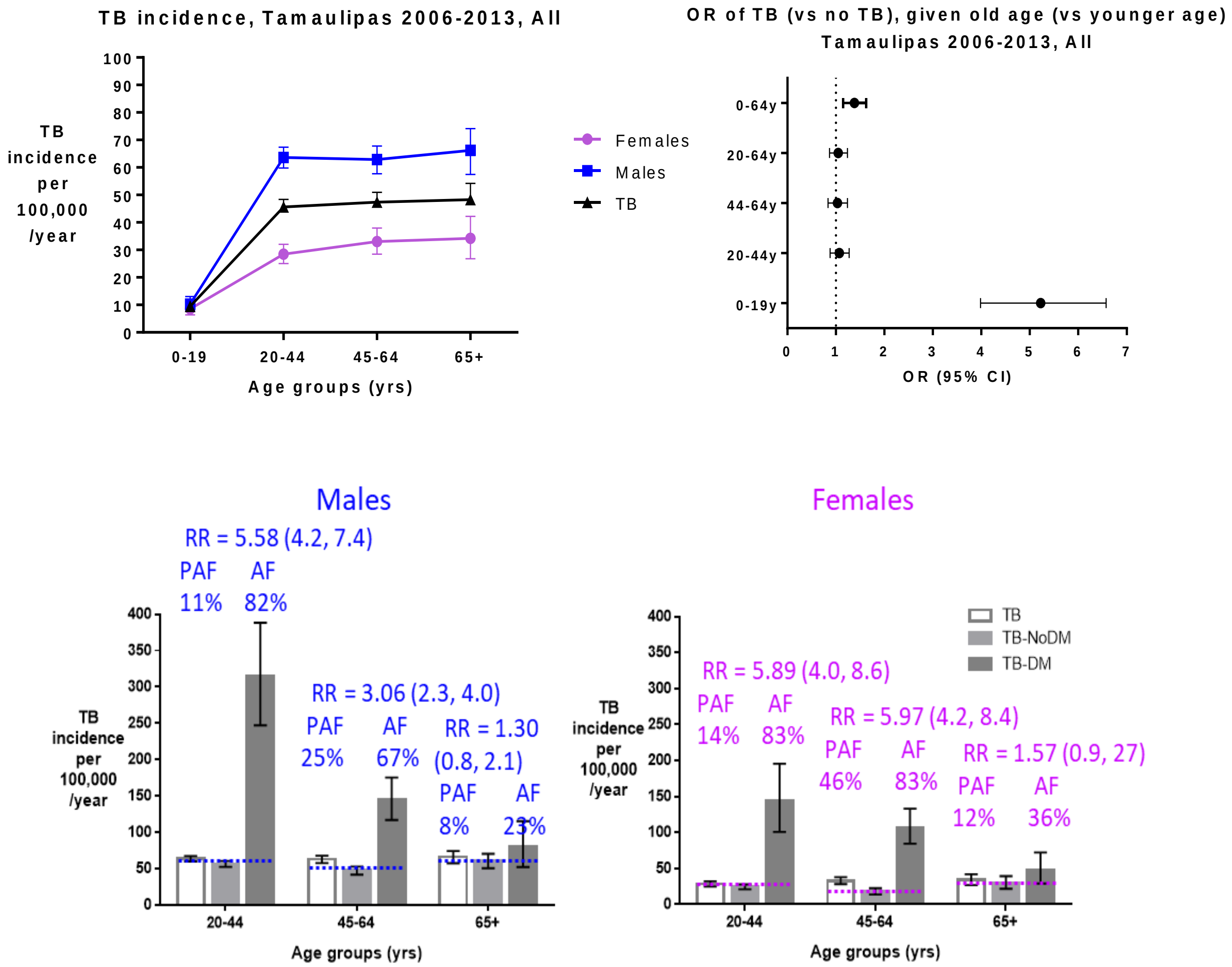

Figure S1. **Sensitivity analysis for results from the validation dataset (Tamaulipas 2006-2013) without removal of participants with HIV, intravenous drug use or excess alcohol**. **A**. Prevalence of TB by sex and age. **B**. OR of TB *vs* no TB in the ELD *vs*: YA (OR 1.06, 95%CI 0.88, 1.27), MAA (OR 1.02, 95%CI 0.84, 1.24), all adults (OR 1.04, 95%CI 0.87, 1.24), or all age groups (OR 1.37, 95%CI 1.48, 1.63). **C**. Relative risk, attributable fraction and population attributable fraction due to T2D, by sex and age group. Symbols and abbreviations: Dots, point estimates; Error bars, 95% confidence intervals; Horizontal dotted line, reference TB prevalence among individuals without T2D for calculation of T2D attributable fraction and population attributable fraction; RR, relative risk; PAF, T2D population attributable risk fraction for TB; AF, T2D attributable risk fraction for TB. CHILD, 0-19 y/o; YA, 20-44y/o; MAA, 45-64 y/o; ELD, ≥65 y/o.

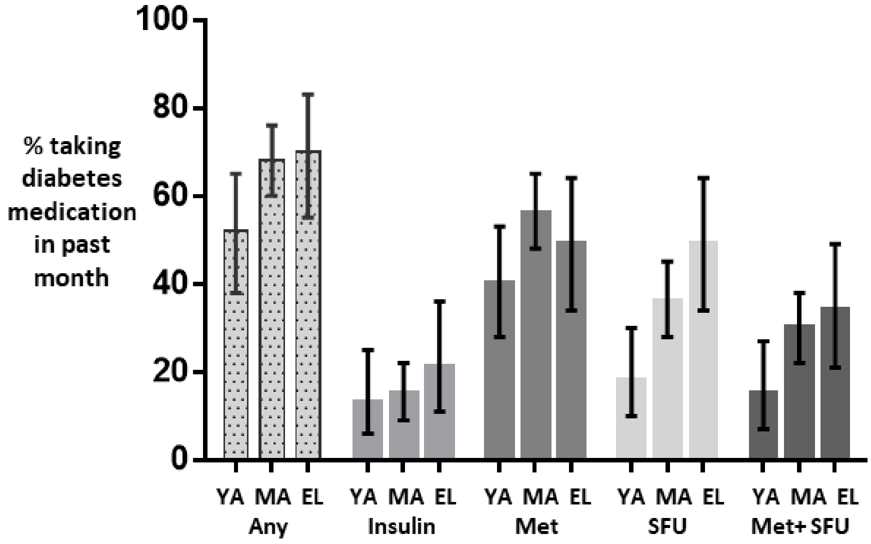

Figure S2. Proportion of diabetes patients using hypoglycemic agents by age group. Few participants also reported use of TZDs, SGLT2I, DPPI or GLP-1 agonists (not shown). Vertical bars, 95% CI; SFU, sulphonylureas; Met, metformin
